# Combining Albumin-Binding Properties and Interaction with Pemetrexed to Improve the Tissue Distribution of Radiofolates

**DOI:** 10.3390/molecules23061465

**Published:** 2018-06-16

**Authors:** Cristina Müller, Patrycja Guzik, Klaudia Siwowska, Susan Cohrs, Raffaella M. Schmid, Roger Schibli

**Affiliations:** 1 Center for Radiopharmaceutical Sciences ETH-PSI-USZ, Paul Scherrer Institute, 5232 Villigen-PSI, Switzerland; patrycja.guzik@psi.ch (P.G.); klaudiasiwowska@gmail.com (K.S.); susan.cohrs@psi.ch (S.C.); raffaella.schmid@bluewin.ch (R.M.S.); roger.schibli@psi.ch (R.S.); 2 Department of Chemistry and Applied Biosciences, ETH Zurich, 8093 Zurich, Switzerland

**Keywords:** pemetrexed, folic acid, radiofolate, albumin-binder, SPECT, ^177^Lu, KB, IGROV-1

## Abstract

Folic-acid-based radioconjugates have been developed for nuclear imaging of folate receptor (FR)-positive tumors; however, high renal uptake was unfavorable in view of a therapeutic application. Previously, it was shown that pre-injection of pemetrexed (PMX) increased the tumor-to-kidney ratio of radiofolates several-fold. In this study, PMX was combined with the currently best performing radiofolate ([^177^Lu]cm13), which is outfitted with an albumin-binding entity. Biodistribution studies were carried out in mice bearing KB or IGROV-1 tumor xenografts, both FR-positive tumor types. SPECT/CT was performed with control mice injected with [^177^Lu]folate only and with mice that received PMX in addition. Control mice showed high uptake of radioactivity in KB and IGROV-1 tumor xenografts, but retention in the kidneys was also high, resulting in tumor-to-kidney ratios of ~0.85 (4 h p.i.) and ~0.60 (24 h p.i.) or ~1.17 (4 h p.i.) and ~1.11 (24 h p.i.) respectively. Pre-injection of PMX improved the tumor-to-kidney ratio to values of ~1.13 (4 h p.i.) and ~0.92 (24 h p.i.) or ~1.79 (4 h p.i.) and ~1.59 (24 h p.i.), respectively, due to reduced uptake in the kidneys. It was found that a second injection of PMX—3 h or 7 h after administration of the radiofolate—improved the tumor-to-kidney ratio further to ~1.03 and ~0.99 or ~1.78 and ~1.62 at 24 h p.i. in KB and IGROV-1 tumor-bearing mice, respectively. SPECT/CT scans readily visualized the tumor xenografts, whereas accumulation of radioactivity in the kidneys was reduced in mice that received PMX. In this study, it was shown that PMX had a positive impact in terms of reducing the kidney uptake of albumin-binding radiofolates; hence, the administration of PMX resulted in ~1.3–1.7-fold higher tumor-to-kidney ratios. This is, however, a rather moderate effect in comparison to the previously shown effect of PMX on conventional radiofolates (without albumin binder), which led to 5–6-fold increased tumor-to-kidney ratios. An explanation for this result may be the different pharmacokinetic profiles of PMX and long-circulating radiofolates, respectively. Despite the promising potential of this concept, it is believed that a clinical translation would be challenging, particularly when PMX had to be injected more than once.

## 1. Introduction

Folic-acid-based radioconjugates have been developed and pre-clinically investigated over the past years for the purpose of nuclear imaging of folate receptor (FR)-positive tumors using Single Photon Emission Computed Tomography (SPECT) or Positron Emission Tomography (PET). Only a very limited number of candidates have been translated to clinical studies, among those [^111^In]DTPA-folate and [^99m^Tc]EC20 (Etarfolatide^TM^) [1,2]. [^99m^Tc]EC20 has been used for imaging of FR-positive malignancies enabling the selection of patients who could potentially profit from FR-targeted chemotherapy [3,4,5]. PET imaging agents using ^18^F-labeled folate tracers are currently under investigation for the same purposes [6,7]. The application of folate radioconjugates for targeted radionuclide therapy would be extremely attractive, given the fact that a large variety of tumor types express the FR, among those several gynecological cancer types, but also other frequently occurring cancer types such as non-small cell (NSC) lung cancer [5,8]. The high accumulation of folic acid radioconjugates in the kidneys has, however, presented a drawback in this regard [9]. A tumor-to-kidney ratio of accumulated activity in the range of 0.1–0.2, as was the case for conventional folate radioconjugates (without albumin binder), prevented the realization of the therapeutic concept completely. Our group has made major efforts to develop concepts that can enable the application of therapeutic folic acid radioconjugates [10].

**Figure 1:**
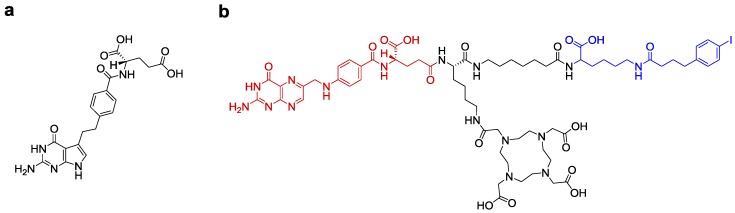
(**a**) Chemical structure of the antifolate pemetrexed (PMX, Alimta^TM^). (**b**) Chemical structure of the most promising albumin-binding DOTA-folate conjugate referred to as cm13 [24]; folic acid (red) serves as the targeting agent; the albumin-binding entity (blue) enables binding to serum albumin and, hence, enhanced residence time in the blood; the DOTA chelator allows stable coordination of ^177^Lu.

We were the first to demonstrate the fact that the antifolate pemetrexed (PMX, Alimta^TM^, Figure 1a [11]) increases the tumor-to-background ratio of accumulated radioactivity when injected one hour prior to the radiofolate [12]. This concept was further investigated in different preclinical settings with a variety of radioligands including [^111^In]DTPA-folate and [^99m^Tc]EC20 that had previously been tested in patients [13,14,15,16]. The “antifolate effect” was reproducible in different animal models including xenograft and syngeneic tumor mouse models [13,14,15]. Hence, it could be shown repeatedly by our group and others that this concept improved the tissue distribution of any radiofolate by reducing retention in the kidneys [17]. We were also able to demonstrate, that the combination of PMX and [^177^Lu]folate can enhance the therapeutic outcome and, in addition, reduce undesired side effects of radiofolates [18]. These findings were based on the radiosensitizing potential of PMX [19,20,21], and the reduction of accumulated activity in the kidneys [12]. Even though this approach revealed to be highly promising, the tumor-to-kidney ratio of accumulated activity was still <1 when using the most promising folate radioconjugates (e.g., DOTA-conjugates [22]). This fact presented a hurdle for therapeutic application of radiofolates as the risk of damage to the kidneys would be high.

More recently, we have pursued another strategy in which we modified the folate conjugate chemically by introducing an albumin-binding entity [23]. This modification was thought to enhance the blood circulation time of the radiofolate ([^177^Lu]cm09) and, hence, improve the tissue distribution profile [23]. Indeed, this concept led to unprecedentedly high tumor-to-kidney ratios of accumulated activity (0.5–0.7 over 5 days p.i.) and had the advantage of avoiding the use of additional medication to reach the desired effect.

By changing the linker entity of the radiofolate, we aimed at further improving the tissue distribution profile [24]. Integration of a short alkane spacer between folic acid and the albumin-binding entity was realized in compound cm13 [24] (Figure 1b). This modification appeared favorable based on a slightly improved tumor-to-kidney ratio of accumulated radioactivity after application of [^177^Lu]cm13 [24].

Based on the observation that PMX improves the tissue distribution of folate radioconjugates the question arose whether the combined application of PMX and [^177^Lu]cm13 would enable a further increase in the tumor-to-kidney ratio of accumulated radioactivity. The aim of this study was, therefore, to combine [^177^Lu]cm13 and PMX in vivo by investigating a potentially positive effect on the tissue distribution of radioactivity. Mice with KB tumor xenografts (cervical cancer type), the most often used mouse model to test folate (radio)conjugates, were employed in the first place for this study. In addition, we investigated mice bearing IGROV-1 tumor xenografts, an ovarian cancer model, which has been used previously for the investigation of folate radioconjugates [15,25]. Biodistribution studies were performed in order to investigate [^177^Lu]cm13 applied alone and combined with PMX injected before (and after) the radiofolate. SPECT/CT imaging was carried out to visualize the anticipated effects.

## 2. Results

### 2.1. Biodistribution Studies

#### 2.1.1. Biodistribution in KB and IGROV-1 Tumor-Bearing Mice

Biodistribution studies were performed at 4 h and 24 h after injection of the [^177^Lu]folate (5 MBq, 1 nmol per mouse) using nude mice bearing KB or IGROV-1 tumor xenografts (Table 1, Table 2 and Table 3). The uptake of [^177^Lu]cm13 in KB tumors was high (22.4 ± 4.50% IA/g) at 4 h p.i. and largely retained over the time of investigation (18.6 ± 6.80% IA/g, 24 h p.i.).

Pre-injection of PMX reduced the uptake in KB tumor (17.6 ± 0.90% IA/g, *p* < 0.001) at 4 h p.i., but had no impact on the tumor uptake at later time points (22.1 ± 3.60% IA/g; 24 h p.i.). At 4 h p.i. retention of radioactivity in the kidneys was significantly reduced when PMX was pre-injected (15.8 ± 2.60% IA/g vs. control mice: 26.5 ± 1.20% IA/g; *p* < 0.0001). The same effect was observed at 24 h p.i. (24.7 ± 5.70% IA/g vs. control mice: 30.9 ± 3.90% IA/g; *p* < 0.01). If PMX was injected for a second time, 3 h or 7 h after administration of the radiofolate, kidney uptake was even more effectively reduced at 24 h p.i. (21.8 ± 0.70% IA/g; *p* < 0.0001 and 21.0 ± 4.70% IA/g; *p* < 0.0001, respectively). In the liver, muscles and salivary glands, PMX did not affect uptake of radiofolate significantly, even though a slight increase in blood activity was seen at 4 h p.i. (~9.13% IA/g vs. control ~7.32% IA/g; *p* > 0.05), but not at later time points (Table 1).

The uptake of the radiofolate in IGROV-1 tumor xenografts was consistently higher (31.5 ± 5.60% IA/g; 4 h p.i. and 37.7 ± 5.10% IA/g, 24 h p.i.) than in KB tumor xenografts. Pre-injection of PMX did not reduce the tumor uptake significantly (*p* > 0.05). A significant difference in tumor accumulated radioactivity was determined, however, between groups of mice that received PMX according to different application schemes. It is not entirely understood why the mice that received PMX only 1 h before the application of [^177^Lu]folate showed the highest tumor uptake (40.7 ± 9.00% IA/g), whereas mice that were injected with PMX 1 h before and 7 h after the radiofolate showed significantly reduced tumor accumulation (32.9 ± 5.30; 24 h p.i.; *p* < 0.001). As compared to control values, PMX reduced the uptake in the kidneys (*p* < 0.001) 4 h after injection of [^177^Lu]folate and in all cases 24 h after injection of the radiofolate (*p* < 0.001). Radioactivity levels in the blood were slightly but not significantly (*p* > 0.05) increased in IGROV-1 tumor-bearing mice that received PMX before the radiofolate (~10.8% IA/g, 4 h p.i.) when compared to control mice (~7.88% IA/g, 4 h p.i.). This effect was still observable at later time points (~1.97% IA/g, 24 h p.i. vs. control mice: ~1.46% IA/g, 24 h p.i.; *p* > 0.05) (Table 1 and Table 3). In the liver, muscles, and salivary glands, PMX did not have a significant effect on the retention of the radiofolate.

#### 2.1.2. Tumor-to-Background Ratios

The tumor-to-background ratios were determined at 4 h and 24 h after injection of [^177^Lu]folate in order to better assess the effects of PMX. This appeared important since the absolute tumor uptake may have varied from mouse to mouse based on the size of the tumor xenograft. In line with the increased radioactivity in the blood 4 h after injection of the [^177^Lu]folate in mice that received PMX, the tumor-to-blood ratios were significantly reduced in KB tumor-bearing mice (1.93 ± 0.18 vs. control mice: 3.05 ± 0.49; *p* < 0.01) as well as in IGROV-1 tumor-bearing mice (2.67 ± 0.58 vs. control mice: 4.05 ± 0.72; *p* < 0.05). At 24 h after injection of the radiofolate, tumor-to-blood ratios of KB tumor-bearing mice were in the same range among the different groups (*p* > 0.05). In IGROV-1 tumor-bearing mice, tumor-to-blood ratios were significantly decreased (*p* < 0.05) at 4 h p.i. in mice that received PMX as well as at 24 h when PMX was applied twice (*p* < 0.05).

**Figure 2:**
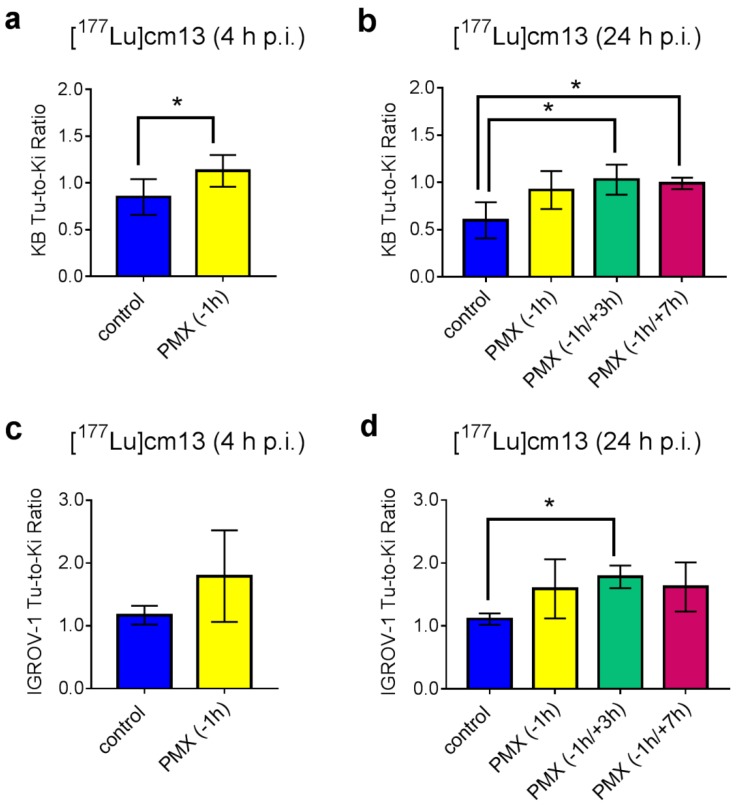
Tumor-to-kidney ratios of mice after injection of [^177^Lu]folate (5 MBq, 1 nmol). (**a**) Tumor-to-kidney ratios of KB tumor-bearing mice 4 h after injection of [^177^Lu]cm13 without pre-injected PMX (blue) or with pre-injected PMX (yellow). (**b**) Tumor-to-kidney ratios of KB tumor-bearing mice 24 h after injection of [^177^Lu]cm13 without pre-injection of PMX (blue) or with pre-injection of PMX 1 h before the radiofolate (yellow) or 1 h before and 3 h (green) or 7 h (red) after injection of the radiofolate. (**c**) Tumor-to-kidney ratios of IGROV-1 tumor-bearing mice 4 h after injection of [^177^Lu]cm13 without pre-injected PMX (blue) or with pre-injected PMX (yellow). (**d**) Tumor-to-kidney ratios of IGROV-1 tumor-bearing mice 24 h after injection of [^177^Lu]cm13 without pre-injection of PMX (blue) or with pre-injection of PMX 1 h before the radiofolate (yellow) or 1 h before and 3 h (green) or 7 h (red) after injection of the radiofolate. Statistically significant values are indicated with asterisks (* *p* ≤ 0.05).

The most important parameter to assess the effect of PMX was undoubtedly the tumor-to-kidney ratio of mice that received PMX compared to the ratio in control mice (Figure 2). In both mouse models, an increased value was observed when PMX was applied. At 4 h after injection of [^177^Lu]folate, the tumor-to-kidney ratio was significantly increased in KB tumor-bearing mice that received PMX (1.13 ± 0.17% IA/g, 4 h p.i.; *p* < 0.05). An increased tumor-to-kidney ratio was also visible in the IGROV-1 tumor mouse model, however, in this case the effect was not significant due to the large standard deviation in the PMX-injected group.

Investigation of the 24 h-time point revealed also consistently increased tumor-to-kidney ratios when PMX was applied. The highest ratios in KB tumor-bearing mice were observed in mice that received PMX 1 h before and 3 h or 7 h after injection of the [^177^Lu]folate (1.03 ± 0.16 and 0.99 ± 0.06, respectively). In IGROV-1 tumor-bearing mice the ratios obtained under these conditions were even higher (1.78 ± 0.18 and 1.62 ± 0.39, respectively) but only significant when PMX was injected 1 h before and 3 h after the radiofolate.

Tumor-to-liver ratios were in the same range for mice injected with [^177^Lu]folate only and mice that received [^177^Lu]folate combined with PMX, independent of which tumor xenograft (KB or IGROV-1) and time point (4 h p.i. or 24 h p.i.) was investigated and whether PMX was injected only once or twice.

### 2.2. In Vivo SPECT/CT Experiments

KB and IGROV-1 tumor-bearing mice were used for SPECT/CT imaging studies 4 h and 24 h after injection of the [^177^Lu]folate only or [^177^Lu]folate in combination with PMX, which was injected before and after the radiofolate. Measurement of the whole mice immediately before the 4 h p.i.-scan revealed that 91–95% of the injected radioactivity (non-decay corrected) was retained in the body independent on whether the mice received PMX. After 24 h, mice that received PMX showed lower radioactivity retention in the body (~62% IA retained in the body, non-decay corrected) as compared to the mice that were injected only with the [^177^Lu]folate (~70% IA retained in the body, non-decay corrected). These data were in line with increased excretion through the kidneys in mice injected with PMX.

#### 2.2.1. SPECT/CT Imaging of KB Tumor-Bearing Mice

**Figure 3:**
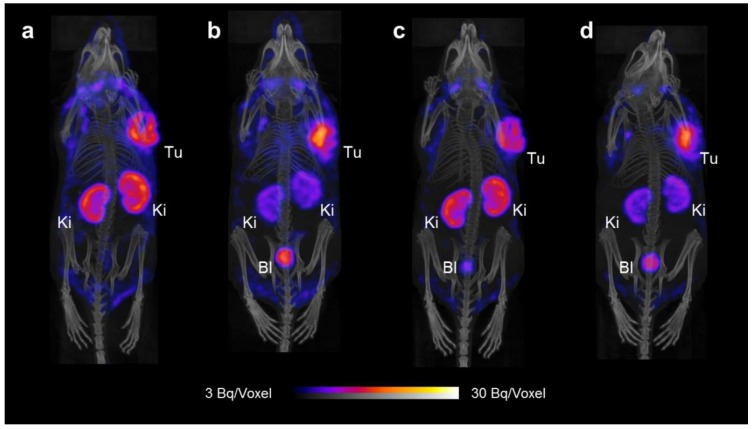
SPECT/CT scans of tumor-bearing mice injected with [^177^Lu]cm13 (25 MBq; 1 nmol) shown as maximum intensity projections (MIPs). (**a**) KB tumor-bearing mouse 4 h after injection of [^177^Lu]cm13. (**b**) KB tumor-bearing mouse 4 h after injection of [^177^Lu]cm13 with PMX injected 1 h before the radiofolate. (**c**) KB tumor-bearing mouse 24 h after injection of [^177^Lu]cm13. (**d**) KB tumor-bearing mouse 24 h after injection of [^177^Lu]cm13 with PMX injected 1 h before and 7 h after the radiofolate. (Tu = KB tumor; Ki = kidney; Bl = urinary bladder).

SPECT/CT scans of KB tumor-bearing mice 4 h and 24 h after injection of the [^177^Lu]folate showed high uptake of radioactivity in the tumor xenografts and accumulation of radioactivity was also observed in the kidneys (Figure 3). Based on a visual analysis, the tumor-to-kidney ratio was ~1 and did not change significantly over the time of investigation up to 24 h p.i. In mice injected with PMX 1 h before the administration of the [^177^Lu]folate, the tumor uptake was slightly increased, while retention of radioactivity in the kidneys was reduced in comparison to the renal uptake observed in control mice. Background activity in blood circulation was reduced over time due to efficient blood clearance of the radiofolate. Other than that, the distribution profile of the radiofolate remained almost identical at 24 h p.i. in the mouse that received PMX a second time 7 h after radiofolate injection.

#### 2.2.2. SPECT/CT Imaging of IGROV-1 Tumor-Bearing Mice

**Figure 4:**
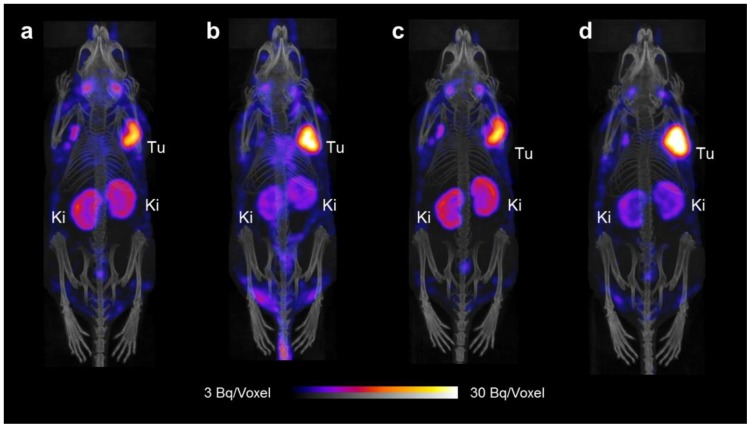
SPECT/CT scans of tumor-bearing mice injected with [^177^Lu]cm13 (25 MBq; 1 nmol) shown as maximum intensity projections (MIPs). (**a**) IGROV-1 tumor-bearing mouse 4 h after injection of [^177^Lu]cm13. (**b**) IGROV-1 tumor-bearing mouse 4 h after injection of [^177^Lu]cm13 with PMX injected 1 h before the radiofolate. (**c**) IGROV-1 tumor-bearing mouse 24 h after injection of [^177^Lu]cm13. (**d**) IGROV-1 tumor-bearing mouse 24 h after injection of [^177^Lu]cm13 with PMX injected 1 h before and 7 h after the radiofolate. (Tu = IGROV-1 tumor; Ki = kidney).

SPECT/CT studies were also performed with IGROV-1 tumor-bearing mice (Figure 4). Radioactivity in the blood pool and heart (background activity) was visible at 4 h p.i. but not anymore at later time points. In line with the biodistribution data, the uptake of the radiofolate was higher in IGROV-1 tumor xenografts than in KB tumor xenografts. Based on visual analysis of the images, the tumor-to-kidney ratios were >1 at 4 h and 24 h after injection of the [^177^Lu]folate. The favorable tissue distribution profile of the [^177^Lu]folate observed in this model was further improved when mice received PMX before (and after) the radiofolate injection. Accumulation of radioactivity in lymph nodes (in the armpit region and next to salivary glands) was more pronounced in IGROV-1 tumor-bearing mice than in mice with KB tumor xenografts.

## 3. Discussion

In this study, we aimed at combining PMX with the currently most promising albumin-binding radiofolate ([^177^Lu]cm13) in order to optimize the tumor-to-kidney ratios further. Our current results were in agreement with those of a preliminary experiment performed with KB tumor-bearing mice and [^177^Lu]cm09, the first DOTA-folate conjugate developed in our group, which was outfitted with an albumin-binding entity (see the Supporting Information of [23]). Although PMX increased the tumor-to-kidney ratio of conventional folate conjugates 5- to 6-fold when injected 1 h prior to the radiofolate [12,15], it was revealed that the effect was much less pronounced when PMX was combined with [^177^Lu]cm09 [23].

In this study, it was shown that PMX was able to increase the tumor-to-kidney ratio of the albumin-binding radiofolate by a factor of ~1.3 at 4 h p.i. and by a factor of 1.5–1.7 at 24 h p.i. in the KB tumor mouse model. The situation was similar in the IGROV-1 tumor model, in which PMX increased the ratio by a factor ~1.5 at 4 h p.i. and by a factor of 1.4–1.6 at 24 h p.i. It was revealed that the distribution of the albumin-binding [^177^Lu]folate benefited from an additional injection of PMX, 3 h or 7 h after the administration of the radiofolate, in order to further reduce the renal uptake and, therewith, increase the tumor-to-kidney ratios.

Even though the accumulation of [^177^Lu]cm13 was clearly higher in IGROV-1 tumor xenografts than in KB tumors, the reported effect was consistent in both types of tumor mouse models. In the case of KB tumor mice the tumor-to-kidney ratios were in the range of ~1.0 when PMX was used whereas these ratios reached values of up to ~1.8 in the case of IGROV-1 tumor-bearing mice. 

When performing this study, it was observed that there were inter-individual differences with regard to the tissue distribution of [^177^Lu]folate. The absolute uptake values determined in this study were also different from previously published values; however, the tumor-to-background ratios were in the same range [24]. The effect of PMX was not exactly the same in each mouse; hence, the interaction between the two drugs appeared to be very sensitive and possibly dependent on other factors.

As already observed in previous studies, the effect of PMX is critically dependent on the time of pre-injection and injected quantity as well as on the amount of injected folate conjugate [13,23]. It is, thus, not surprising that the effect of PMX was less pronounced when combined with long-circulating radiofolates. The albumin-bound fraction of folate radioconjugates is not excreted since albumin is a large protein (>60 kDa) that cannot readily be filtered in the kidneys. Hence, only the free fraction of radiofolates (not bound to albumin) may be affected by pre-injected PMX. Most probably, PMX was excreted already when a major fraction of the radiofolate was still circulating in the blood due to albumin-binding. The fact that repeated injection of PMX was favorable to reduce renal uptake supported the hypothesis that the fast pharmacokinetics of PMX is responsible for the only moderate effect on the distribution profile of the radiofolate. A more sophisticated scheme of PMX application using repeated injections or a slow infusion over the first hours may be successful to reduce renal uptake of albumin-binding radiofolates more effectively. This would, however, be difficult to realize in a clinical setting given the fact that PMX is a chemotherapeutic agent, hence potentially toxic to the patient and not easily upscalable.

In summary, we were able to show, that a “chemical approach,” which refers to the radioligand modification with an albumin-binder and a “pharmacological approach” referring to the pre-injection of PMX can be combined to further improve the tumor-to-kidney ratio of accumulated radioactivity in tumor-bearing mice. This combination led to the best tissue distribution profiles ever obtained with radiometal-based folate conjugates so far. A clinical translation of this approach would be challenging, however, particularly when PMX had to be applied at a dose that induces pharmacological/chemotherapeutic effects.

## 4. Materials and Methods

### 4.1. Preparation of [^177^Lu]Folate

The DOTA–folate conjugate (cm13 [24], herein referred to as “folate”) previously developed by our group was kindly provided by Merck & Cie (Schaffhausen, Switzerland). No-carrier added ^177^Lu was obtained from Isotope Technologies Garching (ITG GmbH, Garching, Germany). Radiolabeling of the folate conjugate was performed in a mixture of HCl (0.05 M) and Na-acetate (0.5 M) at pH 4.5 at 95 °C and an incubation time of 10 min, as previously reported [24]. Quality control of [^177^Lu]folate was carried out using high-performance liquid chromatography (HPLC) as previously reported [24].

### 4.2. Cell Culture

Human KB tumor cells (cervical carcinoma cell line, subclone of HeLa cells, ACC-136) were purchased from the German Collection of Microorganisms and Cell Cultures (DSMZ, Braunschweig, Germany). The human ovarian tumor cell line, IGROV-1, was a kind gift of Dr. Gerrit Jansen, Free University Medical Center Amsterdam, The Netherlands. Both cell lines were cultured under standard conditions (37 °C, humidified atmosphere, 5% CO_2_) in folate-deficient RPMI 1640 medium (FFRPMI, Cell Culture Technologies, Gravesano, Switzerland), supplemented with 10% fetal calf serum, L-glutamine, and antibiotics.

### 4.3. In Vivo Studies

In vivo experiments were approved by the local veterinarian department and conducted in accordance with the Swiss law of animal protection. Athymic female nude mice (CD-1 Foxn-1/nu) were obtained from Charles River Laboratories (Sulzfeld, Germany) at the age of 5–6 weeks. All animals were fed with a folate-deficient rodent diet (ssniff Spezialdiäten GmbH, Soest, Germany). Mice were inoculated with 5 × 10^6^ KB cells or 5 × 10^6^ IGROV-1 cells in 100 µL phosphate buffered saline (PBS) into the subcutis of each shoulder for biodistribution studies. Additional mice were inoculated with the same number of tumor cells into the subcutis of the right shoulder for SPECT/CT imaging studies. For the in vivo scans, mice were anesthetized with a mixture of isoflurane (1.5–2%) and oxygen.

### 4.4. Biodistribution Studies

Biodistribution studies were performed with 4‒5 mice per group, 12‒14 days after KB cell inoculation and 14‒16 days after IGROV-1 tumor cell inoculation. [^177^Lu]folate (5 MBq, 1 nmol/mouse) was diluted in 100 μL PBS and injected into a lateral tail vein (0.05% bovine serum albumin was added to prevent adhesion to the syringe). Pemetrexed (PMX, Alimta^TM^) was diluted in saline (4 mg/mL) and administered at defined time points (0.4 mg per injection) before and after the injection of [^177^Lu]folate. The animals were sacrificed at 4 h and 24 h after administration of the [^177^Lu]folate. Selected tissues and organs were collected, weighed, and radioactivity was measured using a γ-counter (PerkinElmer Wallac Wizard 1480). The results were decay corrected and presented as a percentage of the injected (radio)activity per gram of tissue mass (% IA/g).

### 4.5. Statistics

Biodistribution data were compared using a two-way ANOVA Sidak’s multiple comparisons test (4 h p.i. time point) and a two-way ANOVA Tukey’s multiple comparisons test (24 h p.i. time point; Graph Pad Prism version 7). Tumor-to-background ratios were compared using an unpaired *t*-test with Welch’s correction (4 h p.i. time point) and ordinary one-way ANOVA Tukey’s multiple comparisons test (24 h p.i. time point), respectively. Statistically significant differences were calculated using data based on the average [% IA/g]-values for the radioactivity accumulation in the blood, kidneys, liver, muscle, salivary glands and tumors. Values for tissue uptake that differed significantly from the control group are indicated with asterisks in Table 1, Table 2 and Table 3. Statistically significant differences in tissue uptake among the PMX-pre/post-treated mice were indicated as a footnote to Table 3. All statistically significant differences of tumor-to-background ratios between the groups were indicated with asterisks in Figure 2. Statistically significant values were indicated as follows: ns: *p* > 0.05: * *p* ≤ 0.05; ** *p* ≤ 0.01; *** *p* ≤ 0.001; **** *p* ≤ 0.0001.

### 4.6. SPECT/CT Studies

Imaging studies were performed using a small-animal SPECT/CT camera (NanoSPECT/CT^TM^, Mediso Medical Imaging Systems, Budapest, Hungary). [^177^Lu]folate was injected into the lateral tail vein of tumor-bearing mice (25 MBq, ~1 nmol per mouse). SPECT scans of 38 min duration were performed 4 h and 24 h after injection of the [^177^Lu]folate after CT scans of 7.30 min duration. The images were acquired using Nucline Software (version 1.02, Mediso Ltd., Budapest, Hungary). The reconstruction was performed using HiSPECT software, version 1.4.3049 (Scivis GmbH, Göttingen, Germany). Images were analyzed using VivoQuant software (version 3.0, inviCRO Imaging Services and Software, Boston, US). Gauss post-reconstruction filter (FWHM = 1 mm) was applied twice to the SPECT images, and the scale of radioactivity was set as indicated on the images (minimum value = 3 Bq/voxel to maximum value = 30 Bq/voxel).

## Figures and Tables

**Table 1:** Biodistribution data obtained in KB and IGROV-1 tumor-bearing mice, 4 h after injection of [^177^Lu]folate with and without pre-injected pemetrexed (PMX). Data are shown as % IA/g tissue, representing the average ± S.D.

	[^177^Lu]cm13			
	-	PMX ^(1)^	-	PMX ^(1)^
	4 h p.i.	4 h p.i.	4 h p.i.	4 h p.i.
Tissue	KB	KB	IGROV-1	IGROV-1
	*n* = 5	*n* = 5	*n* = 4	*n* = 4
Blood	7.32 ± 0.85	9.13 ± 0.89	7.88 ± 1.70	10.8 ± 1.43
Lung	4.33 ± 0.44	4.92 ± 0.39	4.47 ± 0.97	6.14 ± 0.97
Spleen	1.51 ± 0.15	1.45 ± 0.06	1.74 ± 0.15	2.16 ± 0.45
Kidneys	26.5 ± 1.20	15.8 ± 2.60 ****	26.9 ± 2.90	16.9 ± 3.10 ***
Stomach	1.37 ± 0.35	1.41 ± 0.19	1.58 ± 0.47	1.83 ± 0.39
Intestines	1.29 ± 0.42	1.38 ± 0.32	1.10 ± 0.18	1.12 ± 0.20
Liver	3.88 ± 0.49	3.31 ± 0.43	3.27 ± 0.49	3.38 ± 0.56
Muscle	1.92 ± 0.25	1.55 ± 0.28	1.17 ± 0.43	1.28 ± 0.38
Bone	1.55 ± 0.10	1.67 ± 0.24	1.38 ± 0.30	1.57 ± 0.25
Tumor	22.4 ± 4.50	17.6 ± 0.90 ***	31.5 ± 5.60	29.2 ± 8.80
Salivary glands	6.78 ± 0.57	5.84 ± 1.25	6.17 ± 0.49	6.18 ± 0.88

**^(1)^** PMX (400 µg/mouse) was injected 1 h prior to the [^177^Lu]folate; Statistical significance is indicated by asterisks (statistically significant difference between uptake in the tissue of control mice and PMX injected mice.) *** *p* ≤ 0.001; **** *p* ≤ 0.0001).

**Table 2:** Biodistribution data obtained in KB tumor-bearing mice, 24 h after injection of [^177^Lu]folate with and without pre- and post-injected pemetrexed (PMX). Data are shown as % IA/g tissue, representing the average ± S.D.

	[^177^Lu]cm13			
	-	PMX ^(1)^	PMX ^(2)^	PMX ^(3)^
	24 h p.i.	24 h p.i.	24 h p.i.	24 h p.i.
Tissue	KB	KB	KB	KB
	*n* = 4	*n* = 5	*n* = 4	*n* = 4
Blood	1.28 ± 0.23	1.39 ± 0.14	1.37 ± 0.17	1.31 ± 0.14
Lung	1.74 ± 0.45	1.69 ± 0.22	1.66 ± 0.15	1.60 ± 0.35
Spleen	0.69 ± 0.14	0.79 ± 0.13	0.80 ± 0.13	0.72 ± 0.15
Kidneys	30.9 ± 3.90	24.7 ± 5.70 **	21.8 ± 0.70 ****	21.0 ± 4.70 ****
Stomach	0.77 ± 0.19	0.70 ± 0.20	0.80 ± 0.14	0.63 ± 0.21
Intestines	0.27 ± 0.07	0.47 ± 0.14	0.30 ± 0.07	0.34 ± 0.06
Liver	2.46 ± 0.16	1.91 ± 0.44	2.04 ± 0.57	2.05 ± 0.05
Muscle	1.56 ± 0.10	1.31 ± 0.25	1.17 ± 0.20	1.50 ± 0.49
Bone	1.09 ± 0.31	0.97 ± 0.15	0.85 ± 0.06	0.95 ± 0.16
Tumor	18.6 ± 6.80	22.1 ± 3.60	22.4 ± 3.20	20.9 ± 5.10
Salivary glands	4.18 ± 0.62	3.71 ± 0.33	3.39 ± 0.30	3.86 ± 0.44

**^(1)^** PMX (400 µg/mouse) was injected 1 h prior to the [^177^Lu]folate; **^(2)^** PMX (twice 400 µg/mouse) was injected 1 h prior to the [^177^Lu]folate and 3 h after [^177^Lu]folate; **^(3)^** PMX (twice 400 µg/mouse) was injected 1 h prior to the [^177^Lu]folate and 7 h after [^177^Lu]folate. Statistical significance is indicated with asterisks (statistically significant difference between uptake in the tissue of control mice and PMX injected mice). ** *p* ≤ 0.01; **** *p* ≤ 0.0001).

**Table 3:** Biodistribution data obtained in IGROV-1 tumor-bearing mice, 24 h after injection of [^177^Lu]folate with and without pre- and post-injected pemetrexed (PMX). Data are shown as % IA/g tissue, representing the average ± S.D.

	[^177^Lu]cm13			
	-	PMX ^(1)^	PMX ^(2)^	PMX ^(3)^
	24 h p.i.	24 h p.i.	24 h p.i.	24 h p.i.
Tissue	IGROV-1	IGROV-1	IGROV-1	IGROV-1
	*n* = 4	*n* = 5	*n* = 5	*n* = 5
Blood	1.46 ± 0.19	1.97 ± 0.15	2.21 ± 0.29	2.08 ± 0.31
Lung	1.88 ± 0.24	2.16 ± 0.23	2.23 ± 0.25	2.21 ± 0.33
Spleen	0.92 ± 0.17	1.19 ± 0.26	1.26 ± 0.33	1.20 ± 0.30
Kidneys	34.0 ± 2.00	26.2 ± 3.50 ***	21.8 ± 2.00 ****	20.8 ± 3.40 **** **^(4)^**
Stomach	0.69 ± 0.19	0.69 ± 0.31	0.78 ± 0.13	0.65 ± 0.17
Intestines	0.49 ± 0.12	0.44 ± 0.08	0.48 ± 0.07	0.43 ± 0.14
Liver	2.75 ± 0.57	2.47 ± 0.51	2.73 ± 0.65	2.41 ± 0.49
Muscle	1.52 ± 0.19	1.36 ± 0.37	1.28 ± 0.52	1.28 ± 0.32
Bone	0.95 ± 0.11	0.96 ± 0.12	1.02 ± 0.17	0.95 ± 0.10
Tumor	37.7 ± 5.10	40.7 ± 9.00	38.6 ± 3.50	32.9 ± 5.30 **^(5)^**
Salivary glands	4.43 ± 0.63	4.28 ± 0.43	3.95 ± 1.03	3.77 ± 0.45

**^(1)^** PMX (400 µg/mouse) was injected 1 h prior to the [^177^Lu]folate; **^(2)^** PMX (twice 400 µg/mouse) was injected 1 h prior to the [^177^Lu]folate and 3 h after [^177^Lu]folate; **^(3)^** PMX (twice 400 µg/mouse) was injected 1 h prior to the [^177^Lu]folate and 7 h after [^177^Lu]folate. **^(4)^** This value was significantly different from the value obtained in mice that received PMX 1 h before the radiofolate; **^(5)^** This value was significantly different from the values obtained in mice that received PMX 1 h before (and 3 h after) the radiofolate. Statistical significance is indicated with asterisks (statistically significant difference between uptake in the tissue of control mice and PMX injected mice. *** *p* ≤ 0.001; **** *p* ≤ 0.0001).
